# Empowering the future: improving community wellbeing and health literacy through outreach and service-learning

**DOI:** 10.3389/fpubh.2024.1441778

**Published:** 2024-08-09

**Authors:** Carolina B. A. Restini, Tracey Weiler, Kirsten A. Porter-Stransky, Peter J. Vollbrecht, Jonathan J. Wisco

**Affiliations:** ^1^Department of Pharmacology and Toxicology, College of Osteopathic Medicine, Michigan State University (Macomb University College-MUC, and Detroit Medical Center-DMC), Clinton Township, MI, United States; ^2^Department of Medical Education, Herbert Wertheim College of Medicine, Florida International University, Miami, FL, United States; ^3^Department of Biomedical Sciences, School of Medicine Greenville, University of South Carolina, Greenville, SC, United States; ^4^Department of Biomedical Sciences, Western Michigan University Homer Stryker M.D. School of Medicine, Kalamazoo, MI, United States; ^5^Department of Anatomy and Neurobiology, Boston University Aram V. Chobanian & Edward Avedisian School of Medicine, Boston, MA, United States

**Keywords:** assessment, student as teacher, community partnership, outreach/engagement, health professions education, scholarship/curriculum development, service-learning, empowerment

## Abstract

Institutions training future healthcare professionals in healthcare and community engagement play a crucial role beyond traditional classroom settings. Recognizing their potential to support under-represented groups and minorities, institutions increasingly encourage engagement with schools and community organizations. However, work remains to advance meaningful and impactful educational outreach and service-learning programs. This manuscript synthesizes the perspectives of a group of medical school educators to discuss developing sustainable programs to engage youth in Science, Technology, Engineering, Math, and Medicine (STEMM) education with a focus on biomedical science. Through near-peer education and service-learning, healthcare students can impart knowledge, provide mentorship, promote enthusiasm for STEMM fields, and nurture health-related self-efficacy within individuals and communities. Collaborative efforts through student-as-teacher approaches bridge health-related disparities and cultivate healthier, more empowered futures for all. We advocate for community outreach strategies that target future health professionals early in their education and support the scholarship of teaching and learning and program evaluation. Successful long-term programs must ensure that results are systematically assessed, measured, and perpetuated. This perspective aims to highlight the role of service learning and community outreach in increasing individual health literacy and fostering an enduring interest in STEMM careers, thereby empowering the next generation of elementary and secondary school students.

## 1 Introduction

For decades, higher education institutions have recognized that they can positively impact their local communities by engaging with individuals, particularly those from under-represented groups, in K-12 schools and other community organizations (1, 2). Strong connections between higher education institutions and the communities they serve are critical to improving community health through education and health workforce training (3, 4). Although the number of healthcare professionals is increasing faster than the global population is growing, the World Health Organization's National Health Workforce Accounts estimates that the health workforce shortage will still be approximately ten million people by 2030 (5). As is often the case, the consequences of this shortage will most likely fall on those underrepresented in science and healthcare fields, including ethnic and racial minorities, those with disabilities, and socioeconomically disadvantaged neighborhoods (6).

We believe that educational outreach and service-learning programs focused on Science, Technology, Engineering, Math, and Medicine (STEMM) have great potential for improving individual and community wellbeing, particularly in these underrepresented demographics. Moreover, community STEMM outreach activities promote interest in STEMM and facilitate the development of problem-solving and communication skills amongst participants, motivating them to engage in post-secondary STEMM education (7, 8). As a result, these individuals are more likely to have STEMM careers, improving their personal and community economic outlook, and helping to address the health workforce shortage (9, 10).

Many STEMM outreach programs focused on the education of K-12 students leverage the efforts of higher education students and faculty as instructors and educators (11–13). These programs impart knowledge and foster enthusiasm for STEMM amongst K-12 students, teachers, and parents/guardians while creating near-peer mentoring relationships that, in the best circumstances, lead to productive connections between communities and health professions institutions. This near-peer mentoring framework allows for education at multiple levels. K-12 students, teachers, and parents learn about healthcare and wellness, while students in higher education develop communication and leadership skills.

**Figure 1:**
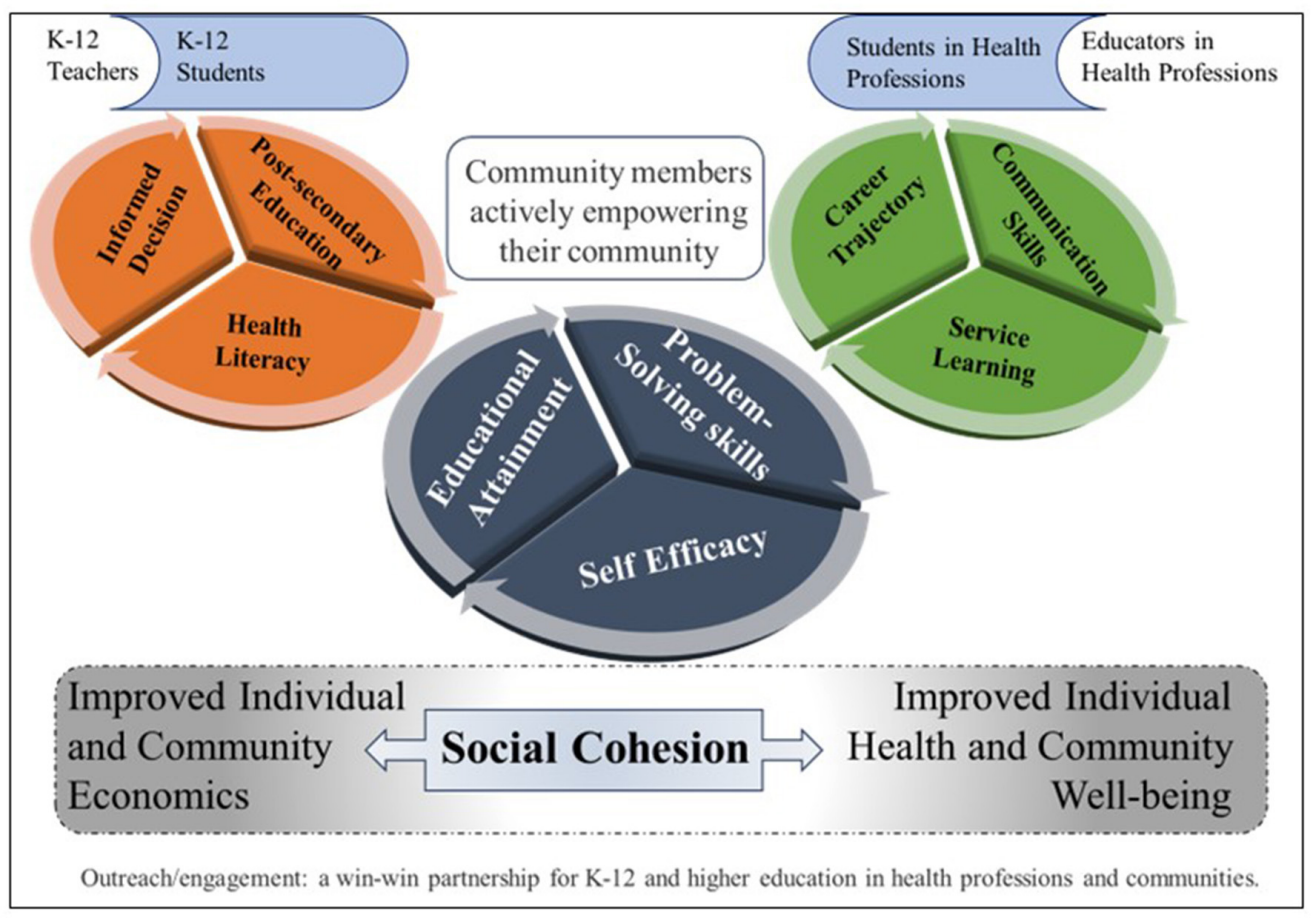
Health professions institutions focus on nurturing health literacy and self-efficacy among communities through service-learning programs for K-12.

On the surface, many of these outreach and service-learning programs are designed to improve public health and STEMM education in the local community, while addressing accreditation standards (14–17). On a deeper level, these initiatives provide education and training to all program participants, empowering them to be change-makers in their families and communities (18). This work requires the investment of time, resources, and collaborative effort from a variety of parties including higher education institutions, students and faculty; K-12 schools, teachers and students; community and healthcare organizations; and families (19). Closer relationships between the community and the higher education institution also enable the institution to develop a better understanding of community needs (Figure 1). This is a win-win collaboration: each participant has something to gain from these programs and something to contribute, resulting in more empowered futures for all participants (11, 12).

Despite the benefits of higher education outreach and service-learning to the community, there are obstacles to establishing ubiquitous university and public school partnerships. First, although the National Academy of Sciences, National Academy of Engineering, and the Institute of Medicine have recommended an increase in engagement by colleges of science in pre-college education (20), outreach and service-learning are not accreditation requirements for all higher education institutions or programs. The Association of American Medical Colleges (AAMC) Liaison Committee on Medical Education (LCME) and the American Osteopathic Association (AOA) Commission on Osteopathic College Accreditation (COCA) require that medical schools incorporate service-learning into the curriculum (21, 22); however, this is not the case for most other STEMM disciplines. Second, with respect to medical school students specifically, the medical school service-learning requirement is not limited to engagement in K-12 STEMM education. Thus, the limited pool of medical students is spread throughout a variety of outreach and service-learning opportunities in addition to K-12 schools, limiting the number available to participate in neighborhood STEMM school programs (23). Finally, the process by which universities and public schools establish partnerships is variable, which could result in unintended, inequitable, or unsustainable programming.

Although these service-learning and student-as-teacher programs represent strategic community educational endeavors, there is only limited evidence of long-term sustainable program success (24–27). The lack of evidence can often be traced back to an absence of programmatic planning, and evaluation (28). Intentional planning and systematic data collection are essential for developing quality, sustainable programs.

This manuscript addresses higher education service-learning and outreach programming through diverse medical school faculty perspectives. Within a common ground, we discuss unique experiences in setting up, evaluating, and supporting these types of programs for K-12 students, educators, schools, families, and local communities (Figure 1).

## 2 K-12 students and community

To achieve positive impacts within their local communities, institutions are implementing outreach and service-learning programs that extend STEMM education beyond the confines of campus lecture halls and clinical settings and into their community environments (13). These community service programs are well-placed to teach scientific principles, model healthy behaviors, and clarify misinformation (29), fostering STEMM and health literacy and ultimately reducing health-related disparities. Education in science, especially regarding concepts of health, holds significant importance in K-12 schools due to its enduring impact on younger learners and their families: A child will live with their body their whole life. This represents opportunities for higher education programs to structure systematic strategies to not only fulfill accreditation standards but also to develop STEMM and health literacy in individual participants, nurturing the seeds of self-efficacy (30) and enhancing their ability to make informed decisions.

The most common barrier to optimal learning in K-12 classrooms is the lack of resources to meet diverse student needs (31, 32). This is especially true in science and health classes where teachers may not have had an academic scientific career (33, 34). Outreach and service-learning programs can address these barriers by providing STEMM role models that demonstrate examples of career pathways to scientific disciplines and careers (35), supplying an external source of short-term expertise and mentoring that emphasizes the “cool factor” of science education (36, 37), and increasing the educator-to-student ratio (38).

Many outreach and service-learning organizations focus on engaging students in science and public health awareness, yet stop short of measuring the effect on K-12 student self-efficacy, self-advocacy, or empowerment (39). Barriers to assessing impact may include an inadequate conception of the goals and outcomes of the program and an insufficient understanding of the difference between programmatic evaluation and outreach/service-learning scholarship based on theoretical and conceptual frameworks for learning. The field of outreach and service-learning would advance significantly if more program directors were supported in scholarly pursuits to study their programs.

## 3 Benefits for future healthcare professionals—a perspective from medical school programs

North American medical schools are engaging in outreach and service-learning, partly as a result of US accreditation agencies that mandate such activities. LCME (21) and COCA (22) state through Element 6.6 and Standard 8, respectively, that programs must provide a strategic plan for educational experiences through community services and scholarly activity. The plans must include cultural competency, health disparities research/scholarly activities, and service to the community in activities that respond to community-identified concerns. To accomplish accreditation requirements, strategies have been reported that place undergraduate medical education (UME) and graduate medical education (GME) students in health and STEMM education settings as paraprofessionals for activities such as clinical case discussions, hands-on clinical skills workshops, class tutoring, and science activities (40). These endeavors can be prospectively extended to other higher education programs with similar accreditation requirements (14–17, 41).

Peer teaching is one such outreach and service-learning strategy, also stated as near-peer teaching (26, 27, 42, 43), peer-assisted learning (44–46), peer tutoring (47), and peer instruction (48, 49). Under faculty guidance, higher education students in the role of teachers are expected to develop learning objectives, prepare materials in advance, create a plan for delivering content through didactics and active learning activities, mentor students, assess their tutees' understanding of the content after delivery, and make necessary adjustments to optimize the program (27, 45). These students may also be involved in research focused on their outreach and service-learning activities (26). They benefit by developing the ability to consolidate and communicate science concepts while becoming humanistic leaders. Consequently, students in healthcare become more confident in their communication, increasing the likelihood that patients will be able to comprehend the information that they provide (46). The development of these educational skills is an important element in the pathway toward career empowerment (45).

Examples of robust outreach and service-learning utilizing the peer teaching model have been put into practice by programs such as Anatomy Academy (50), Brain Explorers (51), and Community-Engaged Teaching and Learning (CETL) (52). Specifically, at Michigan State University, College of Osteopathic Medicine (MSUCOM), CETL was established on the premise that first- and second-year medical students, acting as teachers to contemporaneously closer generations, effectively empower underrepresented high school students and establish better critical learning processes. CETL fosters meaningful community partnerships with high schools surrounding satellite sites of MSUCOM in southeast Michigan through the delivery of biomedical sciences and wellness content. It is uniquely structured to generate research projects. Under faculty mentorship, higher education students learn from and serve their community in continuing educational actions that strengthen university-community connections and empower their participants.

These programs continue in the context of a post-pandemic world in which education is more focused on inclusivity in the classroom and community. We are keenly aware of important lessons learned in the past decade, which have been expressed succinctly by Kemp and colleagues in a recent newsletter publication of the Association of STEMM Pathway and Bridge Programs (53).

## 4 The need to support faculty efforts in outreach and service-learning programs

Sustained and successful service-learning and outreach programs require collaborative long-term partnerships among higher education institutions, community schools/organizations, and educators. Higher education institutions should empower faculty members to mentor and support students engaged in service-learning and outreach. Previous work has revealed that lack of professional incentives, time, and funding are barriers to higher education faculty participation in STEMM outreach and service-learning activities (54). While these barriers are significant, aligning faculty activities with the institution's mission can be helpful. Since most medical schools' mission and vision statements recognize the importance of serving the local community, providing excellent education, and improving health, this alignment is relatively straightforward for medical school faculty. Outreach and service-learning programs directly support these institutional values. Although institutions, by their own mandates, should be supporting faculty in these efforts, faculty may need to self-advocate by articulating the value and alignment of their programs to administrators in order to secure internal funding, time, or other support.

Related to the barriers of lack of time and professional incentives, many faculty are concerned about achieving academic promotion and tenure, in addition to meeting the day-to-day expectations of their positions. We encourage faculty interested in community engagement to find ways to align their STEMM outreach interests with their institution's promotion criteria. Over 30 years ago, Ernest Boyer urged academia to expand the definition of scholarship beyond scientific discovery and to include integration, application/engagement, and the scholarship of teaching and learning (SoTL) (55). In addition, SoTL can generate “promising practices,” an outcome that supports and nurtures ongoing scientific discovery and knowledge generation, humbly recognizing educators' need for continual learning and adaptation to teach learners in different contexts (56). Effective outreach and service-learning programs can include many of Boyer's domains: Outreach and service-learning programs integrate and communicate knowledge, engage communities, and, when done well, incorporate SoTL to maximize the efficacy of the programming. For example, faculty embody their roles as teachers by supervising and mentoring medical students involved in STEMM outreach and service-learning. They can develop research projects assessing learning or systematically evaluating the impact of their outreach or service-learning programs (54, 57).

Furthermore, outreach and service-learning can directly relate to the three central pillars of academia: teaching, research, and service. A conceptual framework on SoTL has been postulated to guide the development and dissemination of scholarly, pedagogical innovations as a path to developing promising practices for teaching and learning in public health (56). However, many institutions do not include specific guidelines for outreach in their promotion and tenure criteria. Nonetheless, there has been progress, with some prominent universities overtly stating outreach as a criterion or a pathway for promotion (19, 57). Outreach activities are not only a service to the community; they can be the focus of a faculty's academic teaching, research, and service missions in meaningful ways.

The outreach and service-learning framework provides opportunities for faculty and medical students to have research experiences (58, 59). The framework embeds multiple components for research, such as the epistemological justification, literature review, and responsible conduct of research. Additionally, faculty can use their outreach and service-learning activities to bolster the broader impacts of their grant proposals (60, 61).

Finally, most STEMM outreach and service-learning programs readily fulfill faculty expectations for community service. We encourage fellow faculty who are passionate about STEMM outreach to align their service-learning or outreach programs with the teaching, research, and service expectations of their academic positions. Documenting accomplishments within STEMM outreach scholarship, teaching, and service can be leveraged as integral components of their academic promotion portfolios. Therefore, although outreach is usually conceptualized as an altruistic activity, it can be mutually empowering for faculty, the institution, and the community.

## 5 Assessment and evaluation

Participation in STEMM outreach continues to gain momentum as academic institutions, individual labs, museums, and other organizations seek to improve STEMM education, access, and literacy. As the number of events, programs, and organizations devoted to this work continues to expand, effective evaluation is critical to be sure that the appropriate programs are receiving support and that those programs that receive support are successful (62–65). Outreach and service-learning evaluation starts with a clear understanding of each program, event, and/or organization's goals. These goals can vary greatly depending on the outreach program, but they must be clearly stated and understood for success to be measured. In their earliest stages, outreach and service-learning events and programs often struggle with this imperative step, either assuming the goals are intuitive or failing to narrow them down appropriately.

Frequent goals of outreach and service-learning include teaching students or increasing public awareness about a particular STEMM topic, providing resources for teachers, or even something as simple as engaging the public with science and public health content. When considering the goals of a program, one should think of goals for each stakeholder, as there may be different goals for each team member. Stakeholders can include the program's target audience and the entire community, in addition to higher education faculty, staff, students, and the institution, amongst others. Evaluations should measure the program's effectiveness in meeting each program goal for each stakeholder group (19).

Evaluation methods can be broad, and the use of a logic model to build and review programs or events is highly encouraged (66–69). The importance of starting with a grounded theoretical or conceptual framework that informs the logic model and research study cannot be overstated (70, 71). In the evaluation of an event or program, various methods can be used, and while it may seem intuitive, care should be taken to collect the appropriate data and select a method that effectively evaluates the program or event's stated goals. This further highlights the importance of setting goals for the program or event during development. These data elements can include demographics, pre-and post-assessments, surveys, interviews, focus groups, or any other type of evaluation. While the logic model outlines assumed causal connections, it may not represent direct cause-and-effect relationships. Given that many factors can influence a program's observed outcomes, it is crucial to consider unintended or unexpected positive, negative, or neutral outcomes. Alternative approaches can be utilized to structure authentic assessments, community development, and ethical principles, such as experiential learning (72) and the change model theory (73). In conclusion, various tools can be used to assess how a clear, long-term goal can be achieved through program engagement, enhancing reasoning skills, problem-solving abilities, teamwork, and communication skills. What is most important is that the chosen evaluation method provides an answer as to whether the program or event is meeting its stated goals.

The ability to evaluate the long-term effects of outreach and service-learning programs is particularly important. This is a wicked problem that needs to be revisited continually (74–76). Long-term tracking of outreach participants is challenging. Even when tracking is done, it is nearly impossible to untangle the large number of variables that may contribute to participants' behaviors and program success (77). Whether or not it leads to scholarship, program evaluation should inform necessary changes that meet community objectives and needs.

## 6 Solidarity in empowerment

Despite the outreach and service-learning requirements of accreditation standards (14–17, 21, 22) and the inclusion of community engagement into the mission, vision, and values statements of many institutions, there has been limited systemic support of research, scholarship, and dissemination of best practices in the areas of STEMM outreach and service-learning. Although there are many governmental and non-governmental organizations focused on STEMM outreach and service-learning, they are typically focused on coordinating and delivering programming to students. The leaders of outreach and service-learning programs require communities of practice to support their efforts (2). These communities of practice provide opportunities for networking between program leaders, which allows for the dissemination of promising practices and collaborative problem-solving among group members. The ability to discuss different methodologies for outreach work, planning, and evaluation is critical for the continuous improvement of these efforts.

The authors are part of two different groups that are filling this gap. The Association of STEMM Pathway and Bridge Programs (ASPBP) was founded in 2022 to support faculty and staff who are delivering STEMM pathways and bridges, including outreach and service-learning around the world (78). Community Outreach, Research & Engagement (CORE) was founded in 2022 as a community of practice specifically for outreach and service-learning in medical education (79). Both groups are focused on networking and leveraging the expertise of the members of the group. Members are invited to share their own innovative programs and outcomes, and the tools they have developed to plan, deliver, and assess them. These presentations to a supportive and engaged audience include robust Q&A sessions that have resulted in identifying best practices. Participants can also seek advice from the group about programs under development. Intentional mission, vision, and values statements guide these groups' evolution within specific frameworks, such as that of justice, equity, diversity, and inclusion. In addition to member networking and presentations, leadership invites external speakers to present on specific topics of interest, fostering the professional development and solidarity of the membership. Over time, members from different institutions have collaborated and presented SOTL projects at regional, national, and international conferences, generalizing best practices for the community of practice (80, 81). An annual peer-reviewed national conference, such as the ASPBP annual conference, can have several positive outcomes. First, it establishes a venue for networking with many like-minded professionals. Second, it provides an opportunity to present research in STEMM pathways and bridges, which has been challenging to do in other venues. Third, it focuses members' attention on collecting data, evaluating progress, and identifying outcomes for their programs, thereby fostering a quality improvement cycle. Fourth, intentional programming at the conference extends faculty development opportunities focused on the STEMM pathway and bridge program creation, delivery, evaluation, and improvement. Non-profit organizations such as ASPBP can also advocate for pathway and bridge programming in STEMM with partner scientific organizations, leveraging the expertise of both. They can collaborate with philanthropic organizations to foster the development of research and scholarship in STEMM pathways and bridge programs through a call for research in this focus area. Finally, they can provide opportunities for volunteer leadership positions, further supporting higher education faculty's promotion and tenure needs. The diverse multi-institutional collaboration to improve the efficacy of pathway and bridge programs around the world influences the trajectory of program participants and empowers them to become agents of change and success in the lives of everyone involved.

## 7 Conclusion

Improving the capacity and quality of the STEMM workforce requires an investment in students early in their educational trajectory. It is incumbent on those working in STEMM to empower students to take control of their future. We believe that early STEMM engagement with K-12 students empowers individuals through health literacy, self-efficacy, and public health awareness. Introduction of K-12 students to individuals in STEMM careers can expose possibilities that might otherwise feel unattainable. In addition to empowering K-12 students, these educational interventions can also empower their families, K-12 educators, the community, and higher education faculty and students, leading to improved economic status, increased diversity of STEMM professionals, and ultimately improved health outcomes (Figure 1). We extend a clarion call to recognize, promote, evaluate, and sustain university and community partnerships that result in improved public health awareness, with a particular interest in understanding how these programs empower all involved.

## Data availability statement

The original contributions presented in the study are included in the article/supplementary material, further inquiries can be directed to the corresponding author.

## Author contributions

CR: Conceptualization, Data curation, Project administration, Supervision, Visualization, Writing – original draft, Writing – review & editing. TW: Writing – original draft, Writing – review & editing. KP-S: Writing – original draft, Writing – review & editing. PV: Writing – original draft, Writing – review & editing. JW: Writing – original draft, Writing – review & editing.
